# Cancer Clinical Trials: Advancing Care, Inspiring Hope

**DOI:** 10.7759/cureus.57891

**Published:** 2024-04-09

**Authors:** Sohilkhan R Pathan, Nirav Asarawala, Kruti B Sharma

**Affiliations:** 1 Clinical Research Services (CRS), Bhanubhai and Madhuben Patel Cardiac Centre, Shree Krishna Hospital and Medical Research Centre, Bhaikaka University, Anand, IND; 2 Medical Oncology, Manibhai Shivabhai Patel Cancer Centre, Shree Krishna Hospital and Medical Research Centre, Anand, IND

**Keywords:** diversity and inclusivity, recruitment challenges, patient outcomes, medical advancements, cancer clinical trials

## Abstract

Cancer clinical trials are instrumental in driving forward medical advancements and enhancing patient outcomes. However, despite their significance, only a small fraction of adult cancer patients, less than one in 20, participate in these trials, indicating significant obstacles in recruitment and engagement. These trials not only assess treatment effectiveness but also serve as vibrant platforms where scientific ingenuity converges with human resilience, fostering a culture of exploration and empowerment. Furthermore, they spur innovation in treatment methods, supportive care, and survivorship strategies, addressing the diverse needs of patients. Moreover, clinical trials prioritize diversity and inclusivity, ensuring that treatments are relevant across various demographic groups and promoting equity in healthcare access. Despite challenges, cancer clinical trials have showcased remarkable resilience and adaptability, particularly in navigating the complexities of the COVID-19 pandemic, underscoring their flexibility and ingenuity. By acknowledging the invaluable contributions of patients and researchers, we recommit to propelling cancer science forward and reshaping the landscape of cancer care for future generations.

## Editorial

Currently, individuals are experiencing extended lifespans due to the outcomes of previous cancer clinical trials. When individuals participate in such trials, they contribute to our understanding of cancer and aid in the enhancement of future cancer care. There are various motivations for joining clinical trials. Cancer patients often participate with the intention of assisting future patients, while those with specific risk factors aim to aid doctors in learning methods for cancer prevention. Healthy volunteers also play a crucial role by assisting doctors in early cancer detection. Ultimately, both cancer patients and healthy volunteers join trials to actively contribute to cancer research and advance scientific knowledge for the benefit of others [1]. 

Fewer than one in 20 adult cancer patients enroll in cancer clinical trials [2]. Timely recruitment of eligible participants is essential for the success of clinical trials, with insufficient accrual being the leading cause for premature termination of both oncology and non‐oncology trials [3]. A meta‐analysis of studies conducted in the United States that examined trial‐decision pathways to elucidate challenges to oncology trial recruitment revealed that in 56% of cases it was due to lack of available trials, 22% did not meet eligibility criteria, 15% of those who were eligible did not enroll, ultimately leading to only 8% of cancer patients participating in clinical trials [4].

In the realm of cancer research, clinical trials stand as beacons of hope, offering not just treatments but also pathways to understanding, empowerment, and even resilience. Beyond their primary aim of evaluating treatment efficacy, cancer clinical trials serve as dynamic arenas where scientific innovation intersects with human courage, fostering a culture of discovery that extends far beyond the confines of the laboratory or hospital. At the heart of every clinical trial lies a partnership between researchers, healthcare professionals, and patients - each bringing their unique expertise, experiences, and aspirations to the table. This collaboration transcends traditional hierarchical structures, fostering a sense of shared purpose and mutual respect. Patients become active participants in their own care, empowered with knowledge and agency to make informed decisions about their treatment journey. Researchers are inspired by the resilience and determination of patients, driving them to push boundaries and explore new frontiers in cancer science.

Moreover, cancer clinical trials are catalysts for innovation not only in treatment modalities but also in supportive care, survivorship, and quality-of-life interventions. Beyond measuring tumor response rates or survival outcomes, trials increasingly incorporate patient-reported outcomes, focusing on aspects such as symptom burden, functional status, and psychosocial well-being. This holistic approach recognizes that cancer is not just a physical disease but also a profound emotional and existential challenge and seeks to address the multidimensional needs of patients throughout their cancer journey. Furthermore, cancer clinical trials serve as crucibles of diversity and inclusivity, transcending geographic, socioeconomic, and cultural boundaries. By actively engaging diverse patient populations, trials not only ensure that treatments are effective and applicable across different demographic groups but also foster a sense of equity and social justice within the broader healthcare landscape. This commitment to diversity is not just a moral imperative but also a scientific necessity, as it enhances the generalizability and impact of trial findings. In the midst of the COVID-19 pandemic, cancer clinical trials have demonstrated remarkable resilience and adaptability, embracing telemedicine, decentralized trial designs, and remote monitoring to ensure continuity of care and patient safety. This rapid transformation underscores the agility and innovation inherent in the clinical trial enterprise, positioning it as a model for healthcare innovation in the digital age.

As we navigate the complexities of cancer clinical trials, let us not lose sight of the human stories that unfold within them - stories of courage, resilience, and hope. Each patient who participates in a trial is not just a data point but a beacon of inspiration, reminding us of the profound impact that research can have on individual lives and the collective human experience. In honoring their contributions, we renew our commitment to advancing cancer science, accelerating innovation, and ultimately, transforming the trajectory of cancer for generations to come.
